# Internal fixation in compound type III fractures presenting after golden period

**DOI:** 10.4103/0019-5413.33683

**Published:** 2007

**Authors:** Quamar Azam, MKA Sherwani, Mazhar Abbas, Rahul Gupta, Naiyer Asif, AB Sabir

**Affiliations:** Department of Orthopedic Surgery, J.N Medical College, Aligarh Muslim University, Aligarh - 202 002, Uttar Pardesh, India

**Keywords:** Early internal fixation, Gustilo's type III fractures, golden period

## Abstract

**Objective:**

Patients often reach the hospital late after passage of golden hours (initial 6 hours) after sustaining high-velocity injuries. The decision of internal fixation in Gustilo's Type IIIA and IIIB fractures becomes a formidable challenge in patients reaching late. The purpose of the present study was to find out if internal fixation could be safely undertaken in these patients.

**Materials and Methods:**

Sixty-three patients, having 70 compound fractures (46 Type IIIA and 24 IIIB), which were internally fixed after 6h but within 24h after injury, were included in the present analysis. Follow-up ranged from 18 to 48 months with mean of 28 months.

**Result:**

Overall infection rate noted was (n = 11) 15.71% (8.7% in IIIA, and 29.16% in IIIB). The difference in deep infection rate between Type IIIA and Type IIIB was found to be statistically significant (*P* value < 0.01). Nonunion was seen in five fractures. Functional evaluation using Katenjian's criteria, showed 62.85% (44 fractures of 70) good to excellent results.

**Conclusion:**

Satisfactory results may be obtained in Gustilo's Type IIIA and IIIB fractures even if fixed after the golden period, provided strict protocol such as aggressive debridement, prophylactic antibiotic coverage, early soft tissue reconstruction and timely bone grafting is followed. The primary coverage of the wound is discouraged.

The aim of fracture treatment is the return of the injured extremity to full function in the shortest period of time. Factors such as contamination of wound, major bone or skin loss and nerve or tendon injuries often associated with Gustilo's compound Type III fractures make this a challenging task. The incidence of infection in cases of intramedullary nailing after external fixator removal is noted to be high.^1^ The external fixator does solve the problem to a great extent, but it often leads to malunion,^2^,^3^ delayed union, loss of reduction and pin track infection. Moreover, the pins act as an obstacle to subsequent wound cover.

Good to excellent results with primary internal fixation in compound fracture following strict protocol have been described.^3^–^6^

The available literature on the subject of internal fixation in compound fractures has not specifically mentioned the exact delay in operation since the time of injury. Only a few articles^3^,^7^,^8^ which exclusively deal with internal fixation in compound Type III fractures are available.

With impaired vascularity in the zone of injury, the body's immune system is compromised. During the first two hours, the host defense works to decrease the overall bacterial load. During the next four hours the number of bacteria remains fairly constant, with the bacteria that are multiplying and those that are being killed by the host defense being about equal. These first six hours are thus called the golden period, because after this period, invading organisms, in the presence of abundant necrotic tissues, replicate in logarithmic fashion to establish a clinical infection. A contaminated wound is considered infected after 12h. The time limit is shorter in a severely contaminated wound with severe soft tissue injury. Internal fixation is advocated within 6-8h in majority of their cases, which is considered the golden period for any surgical intervention.

Unfortunately, in developing countries due to lack of healthcare facilities, ignorance and poverty, patients often reach the hospital late after the few initial precious hours have passed.

In this prospective study of 63 patients (70 fractures), we present our results of internal fixation in Type IIIA and IIIB compound fractures, which were fixed after 6h but within 24h.

## MATERIALS AND METHODS

**Figure 1:**
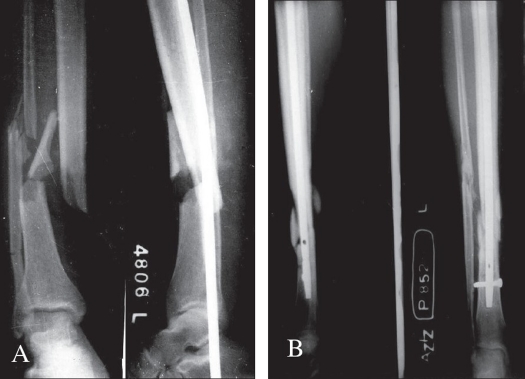
A) Radiograph (anteroposterior and lateral) of a 26-year-old male who sustained Type IIIB comminuted fracture both bone leg. Interlocked nail was done 11 hours after injury. B) Radiograph of the same patient after four months, showing union in progress

**Figure 2:**
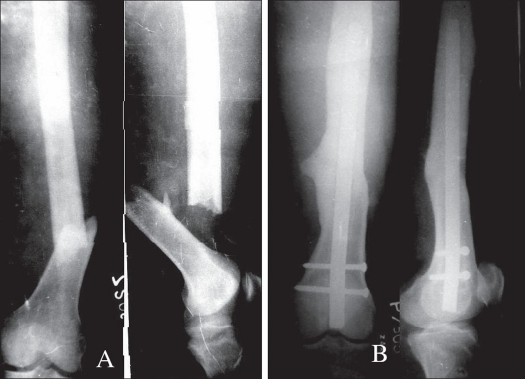
A) Anteroposterior and lateral radiograph of a patient having compound Type IIIB supracondylar fracture femur fixed with intramedullary supracondylar nail after 13h of injury. B) Showing good union after 23 weeks

In this prospective study between February 1996 to October 2000, 63 patients having 70 compound Gustilo Type III fractures (46 IIIA, 24 IIIB) were included. There were 46 males and 17 females, M:F ratio being 2.7:1. Age of the patients ranged from eight years to 65 years with mean age of 37 years. Wounds were classified using Gustilo's classification system.^2^ Only compound Type IIIA and IIIB fractures operated after 6h but within 24h were included in this series [Figures 1 and 2). Patients with compound fracture Type IIIC and those having paraplegia following associated spinal injuries were not included in this study, to avoid bias in the result.

In the emergency room before splinting the extremity, gentle wound toilet with copious amount (3-5 liters) of sterile saline was done and the wound was covered with sterile dressing. All patients received third generation Cephalosporin (1g stat, then 1g six-hourly) and Amikacin (5-7 mg/kg stat, then 15 mg/kg/day in divided doses) for three days. Oral antibiotic was then continued for 10 to 12 days.

The injuries were caused by road traffic accidents in 35 patients (55.55%), industrial accidents in eight (12.69%), fall from height in seven (11.11%), gunshot injuries in five (7.93%), crush injuries in four (6.34%) and others in four patients (6.34%). Associated injuries included ipsilateral and contralateral lower extremity fracture (n=10), fracture of the upper extremity (n=11), clavicle fracture (n=8), thoracic injuries (n=7) and pelvic injuries (n=6).

The different fracture patterns noted in our series were transverse fractures (n=17), oblique and spiral fractures (n=10), comminuted fractures (n=22), segmental fractures (n=12) and intercondylar fractures (n=9). Table 1 details the site of fracture, type of compounding and internal fixation device used. Selection of the implant depended upon the age of the patient, site of fracture and geometry of the fracture.

**Table 1:** Fracture site, Gustilo's type and fixation method used

Fracture site	Gustilo's type		Fixation method

	IIIA	IIIB	
Femur			
Subtrochanteric	2	-	PFN
Diaphyseal	5	3	K-Nail, IL Nail
Condylar- supracondylar	2	1	Multiple K-Wires DCS/SCN
Tibia			
Diaphyseal	13	11	IL Nail, Plate, K-Wire
Condylar	3	-	Buttress plate
Ankle			
Bimalleolar	4	2	Semi-tubular plate, screw, TBW
Humerus			
Diaphyseal	4	2	Plate, K-Wires, IL Nail
Intercondylar	2	1	Semi-tubular/Reconstruction plate
Forearm bones			
Olecranon	2	-	TBW
Monteggia fracture	1	-	Plate
Galeazi fracture	1	-	Plate
Fracture both bone	7	4	Plate, K-Wires, Talwarker Nail

PFN - Proximal femoral nailing, DCS - Dynamic condylar screw, SCN - Supracondylar nail, TBW- Tension band wiring, IL Nail - Interlocking nail

Nine fractures reported within 6-10h of injury while 27, 23 and 11 reported after 10-14h, 14-18h and 18-24h respectively. Two fractures were fixed within 6-10h of injury while 23, 29 and 16 were fixed after 10-14h, 14-18h and 18-24h respectively. The patients were taken for surgical debridement and fixation, on an average within 4.7h (range 2-11h) after reaching the hospital. Besides late presentation other causes of delay in fixation were associated injuries to other vital organ requiring operation (n=13), need to perform additional diagnostic procedure (n=9) and unavailability of operation theater (n=6).

Aggressive debridement that involved exploration of the wound, excision of devitalized tissues and removal of foreign materials, was performed meticulously. In cases (n=42) where the viability of marginal tissue remained doubtful, repeat debridement was undertaken within 48 to 72h. Soft tissue coverage depended on the operating surgeon's appraisal of the wound. Once the wound looked healthy with sprouting granulation tissues early coverage was planned without delay. The average number of surgeries performed was 4.5 (3 to 8) including debridement and removal of implant.

As recommended by Whittle *et al.*,^3^ unreamed nailing was encouraged in our series of patients. However, care was taken that the diameter of the nail for the femur must be at least 10 mm and for the tibia 9 mm. In four cases of the femur and nine cases of tibia reaming was undertaken to attain the said goal. In the remaining 19 diaphyseal fractures (four femur and 15 tibia) unreamed nailing was performed.

In this series we divided all diaphyseal fractures (n=32) of the lower extremities into two subgroups. The first subgroup contained 21 fractures that had bone defects less than 2 cm in length and/or cortical apposition more than 50%. Five of these fractures united after primary internal fixation whereas in the remaining 16 additional procedures like dynamization (n=9), exchange nailing (n=5) and cancellous bone grafting (n=2) were undertaken to promote union. Bone grafting was done in two patients after six months where the butterfly fragment was not in contact with either of the major fragments and did not show sign of union. Dynamization (n=9) was performed after 16-20 weeks when it was assessed radiologically that dynamization will permit compression at fracture site without compromising stability. In another five cases of Group 1 where fracture failed to unite even after 10-12 months, closed exchange nailing after thorough reaming was performed.

The second subgroup contained 11 patients having bone loss more than 2 cm and/or cortical apposition less than 50%. Bone grafting was done in all of them after the wound was fully re-epithelialized. Three of the 11 cases required two bone grafting procedures. In fractures requiring flap, grafting was undertaken only after three to five weeks of successful coverage of wound.

The various wound management techniques used in this series were primary closure (over vacuum suction drain without any tension at the stitch site), delayed closure (n=24) done in three to five days, split-thickness skin graft (5-10 days) and flap cover (within 5 to 15 days). Primary closure was not done in any of Type IIIB fractures. Immediate primary closure (n=18), delayed primary closure (n=6) and split-thickness skin graft (n=12) was done in 36 Type IIIA fractures. Flap cover was performed in 10 cases of Type IIIA and in all the cases of Type IIIB fractures after the appearance of healthy granulation tissue. The gastrosoleus muscle was used as local flap for coverage of open tibial fractures in nine cases.

Active and passive physiotherapy of the joints was encouraged as soon as possible. However, mobilization of patients varied depending on the type of fixation, the requirement of plastic surgery and the presence of other injuries. Follow-up of at least 18 months with a mean of 28 months (range 18-48 months) was available at the time of the final evaluation of the result.

## RESULT

In this study, Katenjian^4^ criteria, that include degree of pain, range of motion and deformity to evaluate functional result of upper extremity and also consider gait while assessing result of lower extremity, have been used. Outcome was divided into Excellent (no pain with normal range of motion in the joint), Good (occasional pain with >75% joint motion and normal gait), Fair (pain with ordinary activities, joint motion >50% and walking with limp) and Poor (constant pain, joint motion <50%, deformity and need of cane/crutch for walking). Deep infection was defined as purulent discharge from the tissue contiguous with the fracture site, which occurred in 11 cases (four Type III A, seven Type III B). Average time required to achieve union for diaphyseal fractures of the femur was 6.5 months (5-13 months), of the tibia 7.8 months (6-18 months), of the humerus 4.5 months (3-7 months) and that of forearm bones 5.6 months (4-9 months). Delayed union was seen in 11 cases. Of these, five fractures which included fracture both bone forearm with Talwalker nail (n=2), fracture shaft of humerus with interlock nail (n=2) and subtrochantric fracture with proximal femoral nail (n=1), united without any intervention. The remaining six fractures where additional procedures were performed to stimulate union included two fractures of the tibia requiring bone graft and four diaphyseal fractures (three tibia and one femur) which required dynamization. Nonunion was noted in five cases, which included two cases of diaphyseal fractures of the femur and three cases of tibia. Closed exchange nailing was performed in all of them to obtain union.

Overall 44 (62.85%), 30 out of 46 (65.21% in IIIA) and 14 out of 24 (58.33% in IIIB) showed good to excellent results [Table 2] in our series which was comparable with the results found in the literature.

**Table 2:** Our results based on Katenjian *et al.*,^8^ system

Gustilo type	Excellent	Good	Fair	Poor
III A	5	25	12	4
III B	2	12	7	3

## DISCUSSION

Davis^5^ performed the first immediate internal fixation following timely initial debridement of open fractures. Many orthopedic surgeons and traumatologists^3^,^4^,^6^,^9^ have since then reported satisfactory results of immediate open reduction and internal fixation for all compound fractures. However, the issue of internal fixation in compound Type III fractures still remains controversial, especially in those fractures presenting late after passage of the initial golden hours. Mc Graw *et al.*,^1^ noted high rate of infection if nailing was done after removal of fixator. Katezian^4^, Zadic^6^ and Yokoyama *et al.*^7^ believed there are definitive advantages of primary internal fixation provided infection could be prevented by careful and radical debridement and use of antibiotics.

The overall rate of deep infection in our study was 15.71% (n=11) (8.7% in Type IIIA and 29.16% in Type IIIB). Infection rate in compound Type III fractures as reported previously in the literature^2^,^3^,^7^,^8^,^10^–^12^ ranged from 4.4-9.09% for Type IIIA fractures and from 23-35% for IIIB fractures. Z-test for proportion was used for statistical analysis and a statistically significant difference in deep infection rate between Type IIIA and IIIB fractures was noted (*P* < 0.01). Antibiotics were continued for 10-12 days in our series, the rationale being that wound heals by this time. However, recent reports^13^ have shown that antibiotics given for three to five days are just as effective in preventing wound infection and have the advantage that if infection develops, it will manifest while the patient is still in the hospital.

Sinclair *et al.*^14^ suggested soft tissue coverage following adequate radical debridement as a single-stage procedure. In our series, of the 11 cases which got infected, in seven cases fixation was performed within 16h of injury and in all of them primary closure was provided after debridement in the same sitting. Though the number of cases was inadequate for statistical analysis, authors feel primary closure should be avoided even in slightest doubt, especially when fixation is delayed and this is in consistence with Gustilo *et al.*,^2^,^15^ Blick *et al.*,^16^ Byrd *et al.,*^17^ and and Patzkins *et al.,*^22^ who support serial debridement and early (3-15 days) soft tissue coverage.

In our series, we noted that the wounds when covered within a week or 10 days after injury were associated with significantly fewer major complications than the wounds that were covered late. This is in consistence with Fischer *et al.*^18^ and Byrd *et al.*^17^ and supports the concept that rich vascularity of early muscle cover is effective in removing microbial contamination and accelerates fracture healing.

Of the 32 diaphyseal fractures (tibia and femur), reaming was performed in 13 cases and unreamed nailing in 19 patients. As the number of cases were less, the authors cannot draw a definite conclusion regarding the outcome in reamed vis-à-vis unreamed nailing. Though the grade of compounding did not influence the decision whether to ream or not the authors feel, excessive reaming in compound fractures must be discouraged, as it renders the endosteal surface ischemic and causes further disruption of medullary blood supply in a bone already denuded of periosteum.^3^ Chapman^19^,^20^ stated that reamed nailing carries an unacceptably high rate of infection. However, Lhowe *et al.,*^21^ concluded that reamed nail provides better fixation decreasing incidence of malunion and also reduces the need of bone grafting.

## CONCLUSION

After having obtained 62.85% good to excellent results the authors strongly feel that internal fixation can be safely undertaken within 24h of injury in compound Type III fractures. Metallic internal fixation, if judiciously performed gives parallel or superior results than external fixator device or delayed internal fixation after removal of external fixator system.

Meticulous and repeated debridement is the key to successful management of the open fractures. Assessment of the viability of damaged muscle in such circumstances is difficult and thus we discourage primary coverage of the wounds at the time of first debridement. However, early wound coverage (within 3-15 days) aids the body in sealing off further entry of bacteria. Furthermore, early flap also enhances healing of fracture by increasing vascularity. Internal fixation reduces hospital stay, achieves better anatomical and functional results and it does not hamper early soft tissue reconstruction.
